# *In Vivo* Mapping of Cortical Columnar Networks in the Monkey with Focal Electrical and Optical Stimulation

**DOI:** 10.3389/fnana.2015.00135

**Published:** 2015-11-16

**Authors:** Anna Wang Roe, Mykyta M. Chernov, Robert M. Friedman, Gang Chen

**Affiliations:** ^1^Interdisciplinary Institute of Neuroscience and Technology, Zhejiang UniversityHangzhou, China; ^2^Department of Psychology, Vanderbilt University, NashvilleTN, USA

**Keywords:** cortical column, optogenetics, microstimulation, fMRI, monkey, optical imaging, optical stimulation, infrared neural stimulation

## Abstract

There are currently largescale efforts to understand the brain as a connection machine. However, there has been little emphasis on understanding connection patterns between functionally specific cortical columns. Here, we review development and application of focal electrical and optical stimulation methods combined with optical imaging and fMRI mapping in the non-human primate. These new approaches, when applied systematically on a large scale, will elucidate functionally specific intra-areal and inter-areal network connection patterns. Such functionally specific network data can provide accurate views of brain network topology.

## A View Of The Primate Brain

**FIGURE 1:**
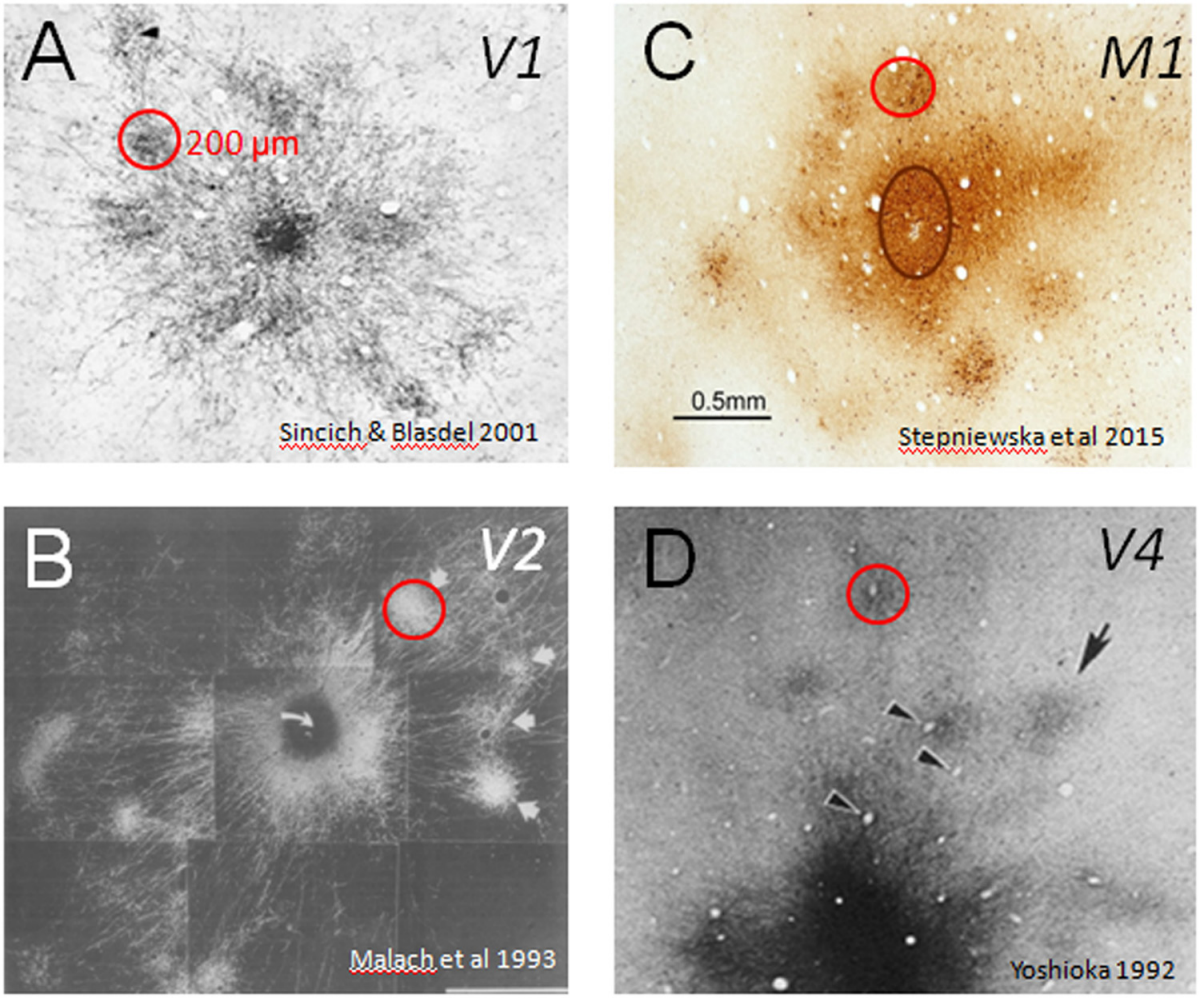
**Cortical modules are connected in specific networks.** Examples of connected columns in **(A)** V1 (Sincich and Blasdel, 2001), **(B)** V2 (Malach et al., 1993), **(C)** M1 (Stepniewska et al., 2015), and **(D)** V4 (Yoshioka et al., 1992). Red circles indicate that column sizes, roughly 200 μm in size, are common to many cortical areas. Scale bar: 0.5 mm applies to all.

In primates (both human and non-human), much of brain volume (up to 80%) is occupied by connections between different parts of the cerebral cortex. Specificity of these connections forms information processing networks that are critical to normal sensory, motor, and cognitive function. Interruption of these connections (e.g., by trauma, stroke) leads to loss or alteration of function. In this sense, the brain can be viewed as a connection machine. There are now largescale projects (termed connectome projects) underway worldwide to study this connection machine (e.g., www.humanconnectomeproject.org, www.brainnetome.org, www.mouseconnectome.org). However, one aspect of these connectome studies that has largely been neglected is the fact that in humans and in primates, cerebral cortex is composed of basic functional columnar units. These units are on the order of a few hundred microns in size and have specific functions (e.g., in visual cortex: processing visual color, shape, depth, or motion information) and are connected in networks with other cortical columns of similar or related functionality (**Figure 1**). Such columnar networks form a basic feature of primate brain architecture. Thus, inherent in understanding the connection machine in primates is developing the ability to systematically map connection patterns between sets of cortical columns.

**FIGURE 2:**
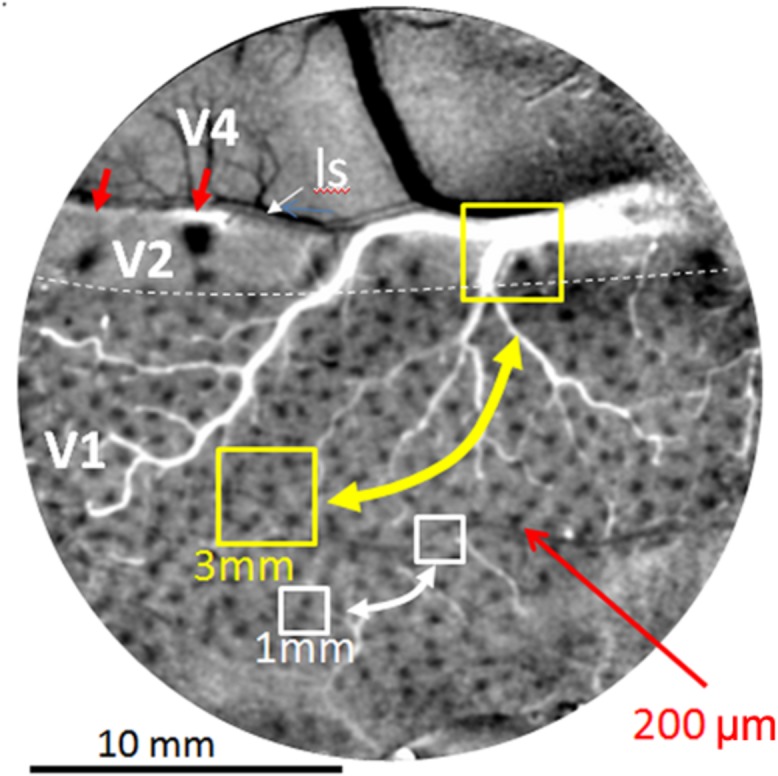
**Optical image of color response seen through an implanted cranial window over macaque monkey visual cortex (areas V1, V2, and V4).** Image obtained by subtracting response to red-green isoluminant grating minus achromatic grating. Arrays of blobs in V1 and thin stripes in V2 (red arrows) reveal size of columnar networks (200 μm). Sizes of voxels used in human fMRI (yellow 3 mm and white 1 mm boxes) include many functional columns; functional resting state connections between these columns (bidirectional arrows) are therefore based on averaged signals and not functionally specific. Small red arrows: two visible thin (color) stripes in V2. ls: lunate sulcus. Dotted white line: V1/V2 border. Scale bar: 10 mm. (Image adapted from Lu and Roe, 2008).

Current human connectomes (based on fMRI study) lack the spatial resolution for examining connection patterns at the columnar scale and therefore lack functional specificity inherent in columnar organization. Individual voxels in these studies represent averages of multiple functional columns (**Figure 2**, yellow box represents 3 mm voxel, white box represents 1 mm voxel); networks of voxels (**Figure 2**, yellow and white bidirectional arrows) therefore represent connections between averages of multiple functional networks. Due to the differential connectivity patterns of individual columns (e.g., color blobs to thin stripes vs. orientation columns to pale/thick stripes, Livingstone and Hubel, 1984; Roe and Ts’o, 1999, 2015; Sincich et al., 2010; Federer et al., 2013), such averages can lead to inaccurate and misleading conclusions about cortical networks.

### Note on ‘Column’ Terminology

Cortical functional units have been described in many areas of visual cortex (V1, V2, V3, V4, MT, inferotemporal cortex) as well as other sensory, parietal, and prefrontal areas and have been termed columns, puffs, blobs, clusters, modules, and domains. Although each term is based on slightly different functional and/or anatomical criteria, they all capture the concept of modularity. For purposes of this review, we will use the term ‘column,’ as it is a generally familiar term and portrays the concepts of: ‘small’ (200–300 μm in size), ‘repeated’ (general architectural unit of the cortex), and ‘functional’ (composed of cluster of neurons which share similar function). This review proposes ways to examine the ‘columnar connectome.’

Note that the concept of a column has of late been controversial, partly because there is not a single accepted definition and because by some criteria cortex is not clearly columnar (e.g., see Rockland, 2010). We use the term ‘column’ less as a physical construct (as its precise nature still needs further study) and more as a term to refer to a modular unit. Our concept of the ‘column’ is that it is a unique site of integration in cerebral cortex, one which is defined by a unique set of (often patchy) inputs and outputs. It is these inputs and outputs that result in a population response that is shared among a cluster of neurons. Although there is controversy regarding what exactly defines the column, the ability to visualize modules of functionally distinct preference is a strong statement that there is modular organization.

## A Columnar Monkey Connectome: A Proposal

We propose to pursue a *columnar connectome in the monkey*; such a connectome would provide a functionally specific understanding of brain networks. While attaining the spatial resolution needed to see columns in humans may be on the horizon (Cheng et al., 2001; Yacoub et al., 2008; Sun et al., 2013), the ability to do so in monkeys is already at hand. Functional imaging at high magnetic fields coupled with use of surface coils has enabled submillimeter resolution in anesthetized squirrel monkeys (Chen et al., 2007), and in awake macaque monkeys can sufficiently distinguish supra-, middle-, and infra-granular laminar specificity and fingerprint V1, V2, and V4 laminar profiles (Chen et al., 2012a,b, 2013; Goense et al., 2012; see also, Olman et al., 2012; Shih et al., 2013; Baek et al., 2015). Moreover, monkeys are excellent animal models for human behavior and disease. They share many commonalities with man including common behavioral repertoires and similar brain structures including the columnar nature of cortical organization (**Table 1**). Furthermore, monkeys can undergo experimental manipulations to answer questions in ways that are not possible in humans. Note that while mouse connectomes have decided advantages (e.g., genetic manipulability), mouse cerebral cortex (except for barrel cortex) is not columnar in the same way as primate cortex. For example, visual orientation selective neurons are not organized in columnar fashion (Metin et al., 1988; Schuett et al., 2002). These differences make certain comparisons less valid for understanding human brain structure, function, and behavior (**Table 1**). A columnar based understanding, we predict, would provide a fundamental component for developing a connectional theory of brain function, one that is not diluted by inaccurate averaged information.

**Table 1:** Comparison of behavioral repertoires and cerebral cortical organization in mouse, monkey, and man.

	Mouse	Monkey	Man
**Vision**			
Fovea, high acuity	No	Yes	Yes
Color vision	No	Yes	Yes
Face perception	No	Yes	Yes
Eye movements	No	Yes	Yes
Visual attention	No	Yes	Yes
**Manual behavior**			
Manual and digit behavior	No	Yes	Yes
Texture on skin	No	Yes	Yes
Limb kinematics	No	Yes	Yes
Shape perception via grasp	No	Yes	Yes
Visuomotor directed grasp	No	Yes	Yes
**Social behavior**			
Language/response alternation	No	Yes	Yes
Social complexity	No	Yes	Yes
**Brain organization**			
Orientation columns	No	Yes	Yes
Large cortex	No	Yes	Yes


## Need For Largescale, Focal Tracing Methods

Anatomical connections can be conducted at columnar scale and have provided some breathtaking views of cortical connection patterns. Perhaps some of the best examples come from monkey V1 where intra-areal patterns of connections have been well documented (e.g., Ts’o et al., 1986; Bosking et al., 1997). There are also a small number of studies on inter-areal columnar connectivity (Livingstone and Hubel, 1984; Roe and Ts’o, 1999; Shmuel et al., 2005; Federer et al., 2013). However, anatomical studies require sacrifice of the animal, and time consuming histological processing and tracer label reconstruction. Within a single animal, connection patterns from only a handful (about five, cf. Zingg et al., 2014) of distinct sites can be examined without compromising the certainty of label identification, placing a limit on the number of different networks one can study in a single brain.

Other methods for studying connections have yet to achieve columnar resolution. *In vivo* diffusion imaging is limited in spatial resolution and therefore accuracy. While it has been useful for some tracts (e.g., Jbabdi et al., 2013), a complete mapping of fiber connections at columnar resolution is unlikely (Thomas et al., 2014). Challenges such as identifying correct fiber courses at points of fiber intersection are still being addressed. Functional and anatomical tract tracing methods in humans using electrical stimulation and imaging (e.g., Lujan et al., 2013) or TMS and imaging (Ruff et al., 2009; Driver et al., 2010; Krieg et al., 2013; Johnen et al., 2015) have been informative, but are also low in spatial resolution. Resting state connectivity in humans (for review, Raichle, 2015), a method based on covariation of hemodynamic response between brain sites, also lacks the spatial resolution for revealing columnar connectivity. While high spatial resolution (mm-scale) resting state connectivity in monkeys can be achieved at high fields (Wang et al., 2013), columnar resolution has yet to be achieved.

Given the constraints of current methods, we have therefore sought to develop new methods to elucidate brain networks systematically, at high spatial resolution, and in a high-throughput manner. In the past few years, we have focused on developing *in vivo* functional tract tracing methods comprised of focal columnar stimulation coupled with optical or fMRI imaging, a method we view as a shortcut toward examining anatomical connectivity. A distinct advantage is that *in vivo* functional tract tracing does not require histological processing and therefore can be conducted repeatedly *in vivo* in the same animal. This enables the study of networks, both at the local and the global scale, from multiple stimulation sites, enabling systematic and large scale collection of connectivity data from a single animal. It is hoped that study of multiple connection networks within single animals can more easily provide data useful for graph theoretical analysis and characterization of network topologies (cf. Sporns, 2010). Note that functional tract tracing is not meant to replace anatomical studies of brain connections, but rather to complement and extend our understanding of the anatomical gold standard.

## Three Functional Tract Tracing Methods

In the following sections, we will describe three functional tract tracing methods that could be used in a high-throughput manner: electrical, optogenetic, and near infrared laser stimulation. Each has its strengths and weaknesses and may be chosen depending on the questions at hand.

### Focal Electrical Stimulation

Electrical stimulation has been a long-standing tool for functional mapping of the brain (Rasmussen and Penfield, 1947). Its uses have spanned mapping cortical organization, distinguishing functional relationships between cortical areas, and applications in brain–machine interfaces (Tolias et al., 2005; Moeller et al., 2008; Stepniewska et al., 2009, 2011; Chase et al., 2012). Importantly, electrical stimulation has been shown to generate or alter normal percepts and behaviors in many species, including human and non-human primates (Salzman et al., 1990; Romo et al., 2000; Godde et al., 2002; Graziano et al., 2002; Murphey and Maunsell, 2007; Tehovnik and Slocum, 2009). However, the relationship between circuits and behaviors activated by electrical stimulation remains poorly understood. This is partially due to a lack of a good understanding of the intra-areal and inter-areal networks activated or modulated via intracortical microstimulation (Bullmore and Sporns, 2009). The power of combining electrical stimulation with functional imaging in a behavioral context has been pioneered in monkeys by Ekstrom et al. (2008, 2009). By conducting fMRI concurrently with stimulation of FEF in the macaque, they demonstrated a contrast-dependent enhancement of visual cortical processing, thereby linking visual behavioral effects with underlying anatomical networks. Developing this approach at columnar resolution would further our understanding of both local and global circuits underlying behavior.

Toward this goal, we have developed methods to map intra-areal and inter-areal circuits using optical imaging combined with focal microstimulation. The underlying viewpoint is that stimulation of a single functional column will lead to activation of other connected columns, both intra-areal and inter-areal. Although electrical stimulation is accompanied by current spread, when delivered at appropriate levels, the effect can remain relatively local. In a two photon study of local neuronal activation in response to well-controlled, low current (5–10 μA) electrical microstimulation, Histed et al. (2009) demonstrated that electrically activated neurons comprised a subset of the neurons within the local vicinity (300 μm) and that movement of the stimulating electrode by 10′s of microns resulted in activation of different subsets of neurons within the same local vicinity. This suggested that, with the right stimulation parameters and precise localization of stimulation location, microstimulation can have quite selective and focal effects.

**FIGURE 3:**
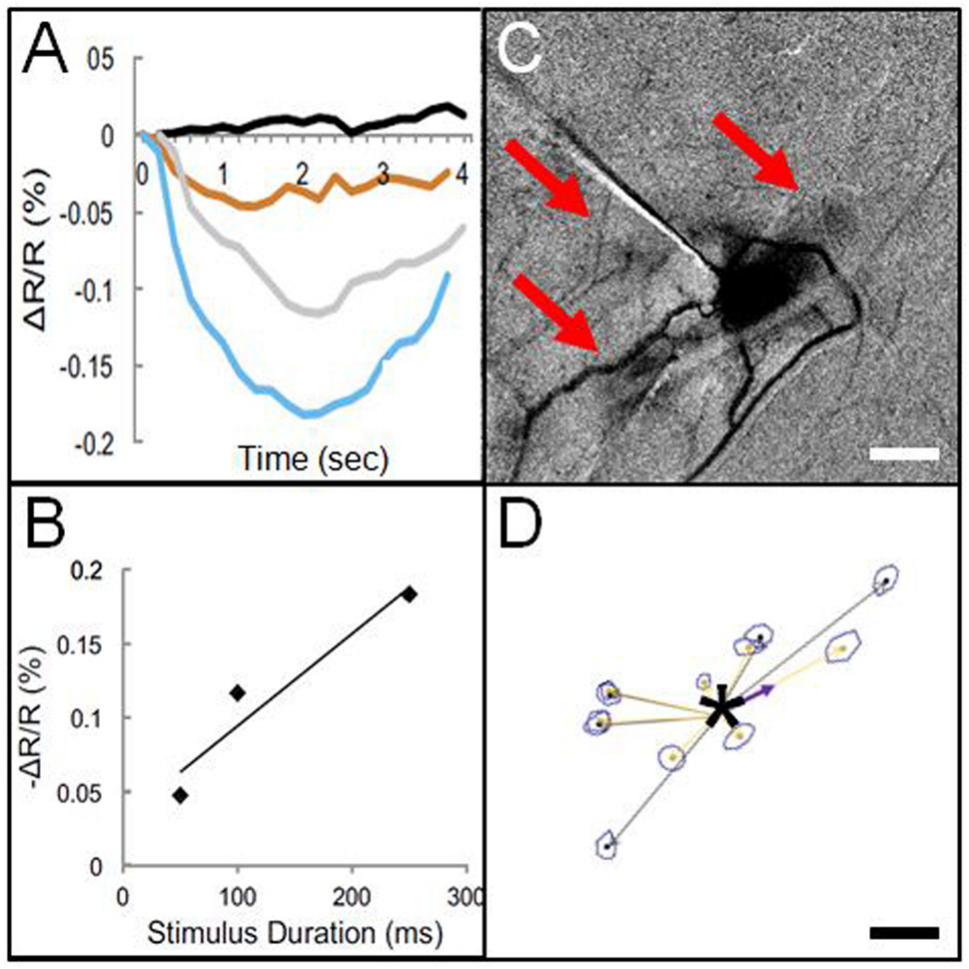
**Optical imaging of focal electrical stimulation in squirrel monkey somatosensory cortex. (A)** Stimulation at 0 s (black), 50 ms (orange), 100 ms (gray), and 250 ms (blue) at 150 μA. *Y* axis: reflectance change dR/R. **(B)** Optical reflectance change increases with stimulus duration (measured at site of stimulation). **(C)** Optical image of local functional connectivity (from Brock et al., 2013) appears similar to **(D)** patches of anatomical label following focal (250 μm sized) injection (asterisk) of BDA tracer (adapted from Negyessy et al., 2013). **(C,D)** are from different animals. Scale bar for **(C,D)**: 1 mm.

This finding by Histed et al. (2009) suggested the feasibility of using electrical microstimulation to map local columnar networks. Encouraged by this study, we attempted functional mapping with electrical microstimulation in monkey somatosensory cortex. As electrical stimulation can produce either excitatory or suppressive effects depending on the stimulation intensity, it was vitally important to characterize stimulus amplitudes by systematically characterizing the stimulation parameter space (e.g., intensity, duration, laminar location, cf. Tehovnik et al., 2004, 2006, 2009). Using trains of biphasic pulses (typically 200 Hz, 0.4 ms pulse duration), and varying intensity by testing different current amplitudes (10–300 μA) and pulse numbers (1, 13, 26, 63 pulses, resulting in total train durations of 0.4, 50, 100, 250 ms), we examined the effect of stimulation intensity on neuronal firing and hemodynamic signal as measured with intrinsic signal optical imaging. With chosen electrical stimulation parameters (25 μA, 250 Hz, 100 ms), the imaged hemodynamic response to electrical stimulation mimicked that of tactile stimulation (**Figure 3C**); both revealed a focal 1 mm activation site consistent with a single digit representation. Increasing intensity of stimulation [either current amplitude (**Figure 3A**) or number of pulses, (**Figure 3B**)] led to increasing magnitudes of imaged reflectance change. Importantly, stimulation (e.g., 200 μA for 250 ms) elicited not only a focal activation at the site of stimulation but other focal activations within 1–2 mm of the stimulated site (**Figure 3C**). This activation pattern is very similar in appearance to typical *intra-areal columnar networks* revealed by anatomical tracer studies (**Figures 1** and **3D**). Direct confirmation of this anatomical correspondence is currently in progress.

**FIGURE 4:**
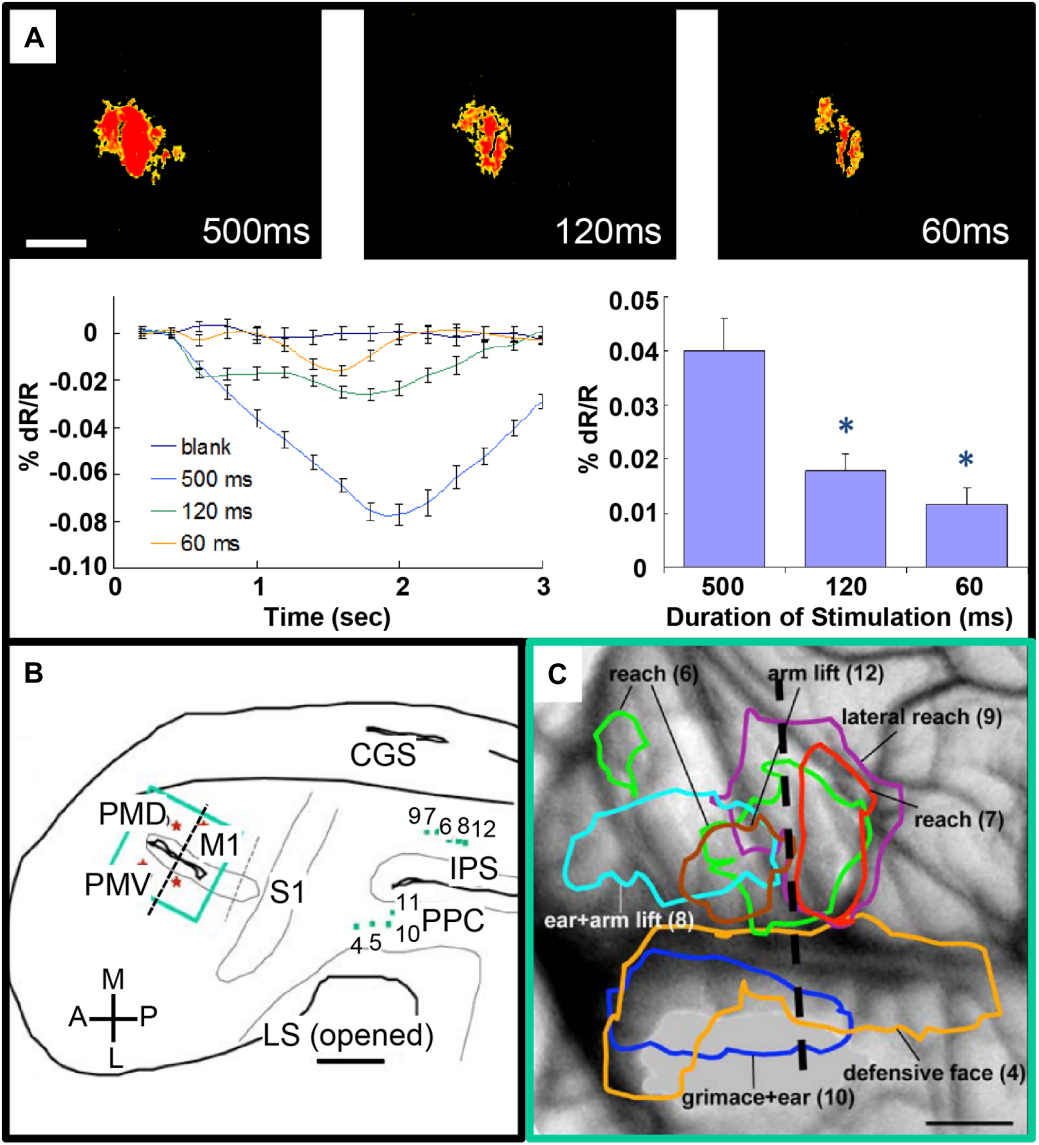
**Intrinsic motor cortex activity to different durations of electrical stimulation of PPC. (A)** Top: three optical images (only significant pixels shown in red, *t*-test ^∗^*p* < 0.01) in response to electrical stimulation (400 μA, 0.1 ms biphasic, 300 Hz) of the dorsal PPC that evoked a lateral reaching movement (left: 500 ms, middle: 120 ms, right: 60 ms stimulation duration). Below: intrinsic signal time courses (left) and peak magnitudes (right). **(B)** Reconstruction of hemisphere. Green dots: stimulation sites in PPC. Green rectangle: imaging field of view centered over motor and premotor cortex. **(C)** Areas of activation in M1, PVD and PMV elicited by electrical stimulation of PPC. Colored outlines: areas activated during face movements (lateral) and forelimb movements (medial). Scale bars: 1 mm. (From Stepniewska et al., 2011).

There is also potential for this approach to be used for mapping *inter-areal columnar connections*. Such an approach has been used to examine connections between motor and parietal areas in the prosimian primate the Galago (Stepniewska et al., 2011). Previous studies have shown that stimulation of parietal cortex at selected sites produces classes of complex behaviors (such as defensive postures, feeding, grooming; Cooke et al., 2003; Stepniewska et al., 2009). To understand the anatomical networks underlying these motor behaviors, optical imaging was used to map motor cortex during stimulation of behaviorally characterized parietal sites. Activations in motor cortex following parietal stimulation were intensity-dependent (**Figure 4A**) and revealed site-dependent differential topography (**Figures 4B,C**). In another study, Arsenault et al. (2014) mapped the functional consequences of stimulating ventral tegmental area with fMRI as well evaluating its behavioral effects and found that free choice behavior could be dramatically altered via widespread activation of the dopaminergic reward system. These studies suggest the possibility of simultaneously evoking or modulating a behavioral effect and mapping the underlying inter-areal circuit.

### Focal Optical Stimulation

#### Advantages

2015 has been designated by the United Nations as the International Year of Light. We owe this to pioneers such as Nobel Laureates in Physics 2009 Charles Kao, Willard Boyle, and George Smith who were innovators in the development of fiber optics and CCD chips. These technologies enabled many advances in optical engineering and in medicine, including technologies for brain stimulation. There are several advantages to replacing wires with light. The most important advantage of this method over electrical stimulation is that it is not encumbered by current spread, making its effect focal. The volume of affected neural tissue is determined by the light wavelength and size of the delivery fiber optic. By selecting optical fibers with illumination spot sizes of 100 μm to 1 mm in diameter, activation of single to several cortical columns can be achieved (Cayce et al., 2011, 2013). Fiber optics can also be selected for stimulation at the brain surface without direct contact to neural tissue and can be easily targeted to specific cortical locations. Deep tissue stimulation via insertion of fiber optics into deep structures is also possible (e.g., Gerits et al., 2012). Another advantage of using light is that it is not accompanied by electrical stimulation artifact, making it readily compatible with electrical recordings. Also, light is easily applied within magnetic fields, making it useful for functional tracing in the MRI.

#### Stimulation through Optical Windows

Optical stimulation techniques can now be introduced via optical windows on the brain. In monkeys, both in anesthetized and awake behaving states, such windows offer the opportunity to probe the brain with multiple techniques. These windows permit multiscale study in individual animals, using methods such as behavioral study, fMRI, optical imaging, electrophysiology, focal stimulation (electrical, optogenetic near infrared laser stimulation), and the study of anatomical connectivity via targeted tracer injections through the window.

### Infrared Neural Stimulation (INS)

The use of infrared wavelength light to evoke neural response was developed by Duco Jansen and colleagues at Vanderbilt University (Wells et al., 2005a,b, 2007a,b). First developed for stimulation of peripheral nerve, this effect is mediated via heat transient induced changes (absorption of infrared light by water) in membrane capacitance and protein conformation (Wells et al., 2007a; Shapiro et al., 2012). The transfer of energy is related to native resonance of water molecules: the better the match between stimulation wavelength and resonant frequency of water, the greater the energy transfer, and therefore, the greater the resulting heat transient; as a corollary, the greater the transfer, the less tissue penetration. Thus, Jansen and colleagues identified a stimulation wavelength (1.875 μm) that resulted in reasonable energy transfer and reasonable tissue penetration of 200–300 μms. When this near infrared wavelength light is presented in brief (0.25 ms) pulses, the transients lead to membrane depolarization and resulting action potentials (see Chernov and Roe, 2014a for review). In contrast to optogenetic approaches, this method is not dependent on viral infection and is thus more amenable to human application; however, unlike optogenetics, this method does not target specific cell types.

#### Infrared Neural Stimulation Intensity Predicts Magnitude of Cortical Response

**FIGURE 5:**
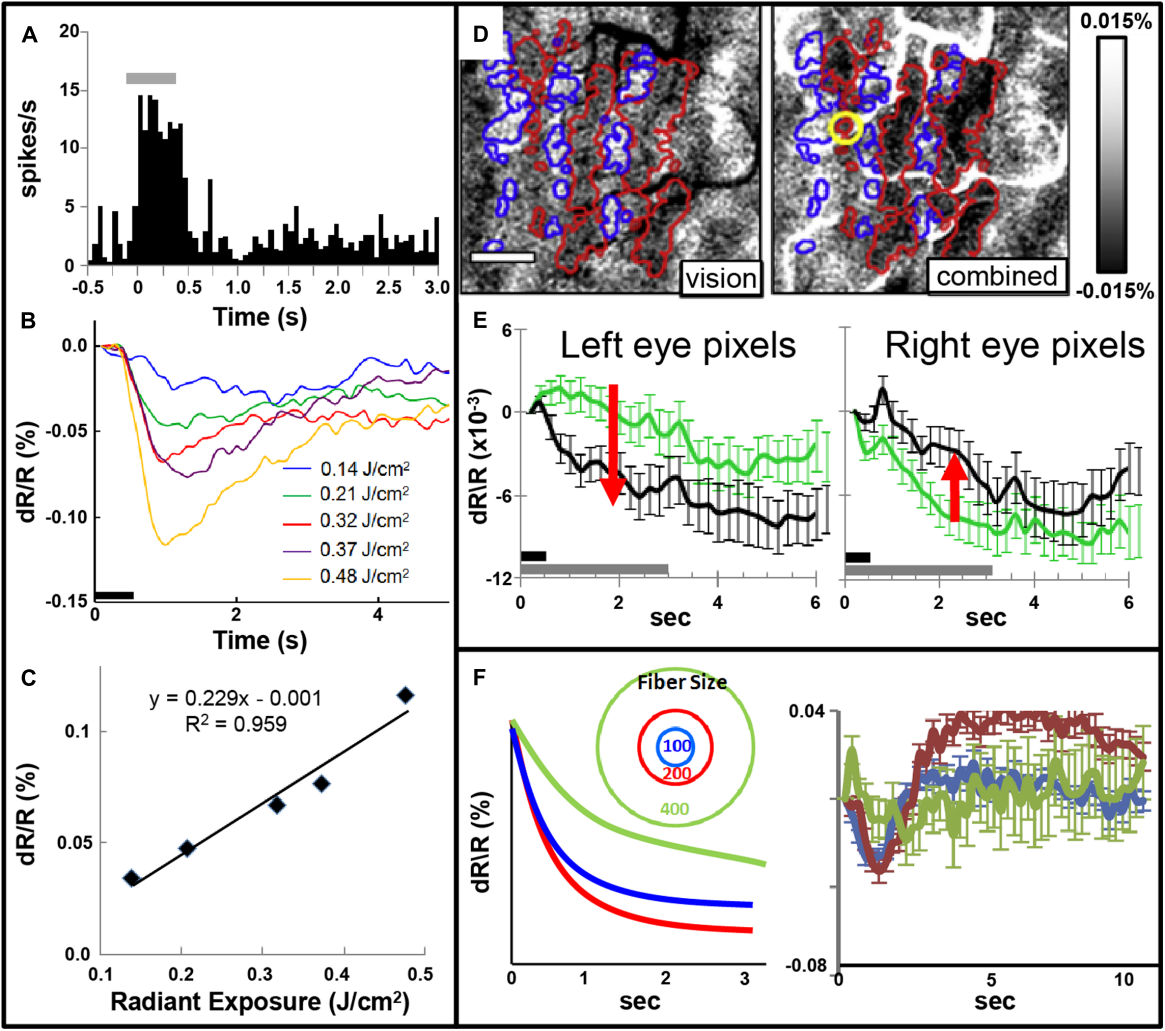
**(A)** Post-stimulus time histogram of neural response evoked by INS stimulation (gray bar). **(B,C)** Increased INS radiant exposure leads to an increase in intrinsic signal magnitude. **(B)** Time course of signal for different radiant exposures. **(C)** Radiant exposure vs. peak amplitude of the intrinsic signal. Relationship fit with a linear equation. (Laser parameters: λ = 1.875 μm, 250 μs pulses at 200 Hz for 500 ms, 400 μm fiber; **D,E**) INS potentiates response to visual stimulation. **(D)** Red and blue outlines demarcate left and right: ocular dominance columns, respectively. Left: OD map generated by subtraction of left minus right eye. Right: INS applied to left eye column during visual stimulation leads to relative enhancement of left eye columns (darkening in red outlined areas) and relative suppression of right eye columns (lightening in blue outlined areas). Scale bar: 1 mm. **(E)** This is observed by examining averaged time courses from pixels in left eye (left panel, red arrow indicates increase in optical signal reflectance) and right eye (right panel, red arrow indicates decline in optical signal reflectance) columns. Dark gray bar: INS stimulation period. Light gray bar: visual stimulation period. Error bars: SEM. (INS parameters: λ = 1.875 μm, 1.3 J/cm^2^, 250 μs pulses at 200 Hz for 500 ms, 100 μm fiber). *Y* axis: dR/R. **(F)** Effect of fiber size. Left: Schematic displaying 100, 200, and 400 μm diameter fibers. Predicted intrinsic signal responses: 200 μm fiber (red) produces larger enhancement than 100 μm fiber (blue). However, 400 μm fiber impinges on domains of other eye and leads to relative suppression (green). Right: actual data is consistent with prediction. (**A–C** from Cayce et al., 2011, **D–F** from Cayce et al., 2013).

Focal INS stimulation induces neural response as assessed with electrophysiology, optical imaging, and BOLD fMRI imaging. Magnitude of this response is related to the intensity of stimulation (**Figures 5A–C**). This focal stimulation can elicit functionally specific effects related to columnar organization. In visual cortex, application of focal stimulation to single ocular dominance columns in V1 can, in combination with visual stimulation, enhance response of shared eye ocular dominance columns (**Figures 5D,E**), suggesting a selective modulation of functionally specific intra-areal networks in V1. Similar modulatory results, by electrical stimulation, have been obtained by Ohayon et al. (2013).

Note that, similar to inhibitory effects of electrical stimulation, optical stimulation can also lead to relative suppression of cortical response. We have observed this using fiber optics of different diameters aimed at cortical columns. We find a U-shaped response where small fibers (100 μm) evoke a response weaker than 200 μm fibers and 400 μm or 1 mm diameter fibers lead to relatively suppressed effects (**Figure 5F**). We interpret this as the larger fibers recruiting additional inhibitory circuits in the surround which change the balance of ongoing intra-areal networks and result in relative suppression.

#### Functional Tract Tracing with INS

**FIGURE 6:**
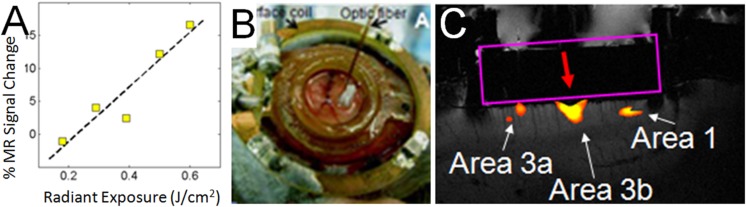
**Functional tract tracing with INS stimulation and fMRI. (A)** Increasing INS stimulation intensity produces increasing BOLD response. **(B)** View of 400 μm fiber optic applied to optical window at D2 location (as determined by optical imaging in different session) in area 3b of somatosensory cortex in a squirrel monkey. The monkey was imaged at 9.4T (Varian Inova Magnet) with a 3 cm surface coil surrounding the optical window. **(C)** Red arrow indicates the location of apposed optical fiber. INS stimulation (0.4 J/cm^2^, pulsed trains applied in blocks of 30 s ON, 30 s OFF) at this site produces strong BOLD response at fiber tip as well as at corresponding topographic locations in area 3a and area 1. Coronal slice: voxel 625 μm × 625 μm × 1000 μm.

Having characterized the effects of INS stimulation, we next examined whether it could be used as a tool for functional tract tracing. To further motivate the use of this approach, we examined the compatibility of INS with fMRI (Chen et al., 2012c). With fMRI, similar to hemodynamic signals measured with optical imaging, the relationship of INS intensity is proportional to hemodynamic BOLD response (**Figure 6A**). Preliminary studies in squirrel monkeys implanted with optical chambers over somatosensory cortex (imaged with surface coil in a 9.4T Varian magnet) have shown that targeted stimulation of single digit locations (e.g., D2 tip) via apposition of the stimulating fiber optic to the cortical surface at one somatosensory cortical location (**Figure 6B**) leads to significant BOLD response in topographically appropriate locations in nearby areas (**Figure 6C**). When imaged in coronal slices, activations appear localized to superficial or middle layers of cortical laminae, suggesting that this resolution is sufficient for superficial, middle, or deep laminar localization (**Figure 6C**). INS combined with fMRI thus provides the opportunity to examine intra- and inter-areal connection patterns of a stimulated site, as well as identify resulting laminar profiles, that will be useful for interpretation of feedforward or feedback connection patterns.

#### Behavioral Effects with INS

There is a long history of focal electrical stimulation producing highly specific behavioral effects (e.g., for motion direction percepts, Salzman et al., 1990; for tactile frequency percepts, Romo et al., 2000). Can INS also be used for similar applications? As a first attempt to examine this possibility, we tested whether focal INS stimulation in visual cortex of macaque monkeys could induce percepts of light spots (phosphenes). In awake fixating monkeys implanted with optical chambers over visual cortex (V1, V2, V4), we used intrinsic signal optical imaging to map the visuotopic representation within the optical chamber (Tanigawa et al., 2010). We then applied INS stimulation to selected sites. As predicted, the induced percept of a light spot led to reliable eye saccades to the predicted site with the appropriate 300 ms latency for eye movements (Roe et al., 2013). This simple baseline test suggests that INS can be used to modulate behavioral outcome in ways similar to previous electrical stimulation studies. We predict that targeting stimulation to specific columnar locations will enable more selective behavioral effects than larger, multi-columnar activations.

#### Optogenetic Stimulation

The widespread use of optogenetics in neuroscience owes its thanks to the pioneering work of Karl Diesseroth, Edward Boyden, and their colleagues at Stanford and MIT. The key advantage of optogenetics is the ability to selectively target specific cell types. Neuronal response to optical stimulation is achieved by incorporation of light-sensitive rhodopsin molecules via molecular genetic techniques. They can be triggered with millisecond precision (Han and Boyden, 2007) and can lead to either excitatory (Boyden et al., 2005) or suppressive neuronal effects (Chow et al., 2010). Channelrhodopsin-2 (ChR2) is a light sensitive cation channel that leads to depolarization of neurons. Hyperpolarization can be achieved using halorhodopsin (a light-activated chloride pump) or archaerhodopsin (Arch; a light-activated proton pump; Boyden et al., 2005; Han and Boyden, 2007). In mice, this has been a boon to circuit dissection underlying behavior and disease (for review see Bernstein and Boyden, 2011).

In monkeys, however, progress has been slower. Initial studies in primates demonstrated feasibility of expression and behavioral effect (Han et al., 2009, 2011; Diester et al., 2011; Cavanaugh et al., 2012; Galvan et al., 2012; Jazayeri et al., 2012). Surprisingly, unlike electrical stimulation, optogenetic stimulation appears to have more subtle effects. Rather than eliciting robust motor responses, optogenetic activation appears to modulate activity, either ongoing neural activity or to activity induced by electrical stimulation. Examples include optogenetic inactivation of monkey superior colliculus which leads to saccadic eye movement deficits (Cavanaugh et al., 2012), optogenetic activation in FEF which has subthreshold eye movement effects when paired with electrical stimulation (Ohayon et al., 2013), and optogenetic activation in parietal cortex which lead to modulation of salience maps (Dai et al., 2014). There may be multiple reasons for the apparent ‘weaker’ effect of optogenetic stimulation, such as differences in the mechanism of activation, differences in neural populations being activated, or the small number of cells effectively transfected.

Yet another potential cause relates to limitations in methodology. These limitations include inconsistencies in viral delivery, robustness of viral infection and expression, accuracy of light delivery, and reliability of *in vivo* assessments of neural or behavioral effectiveness. One approach for improving optogenetic technology in monkeys is the use of optical windows. In the primate, the presence of heavy dura mater has necessitated use of large bore injectrodes, electrodes, and optrodes (e.g., Diester et al., 2011) which can damage cortex; the opacity of dura mater also leads to uncertainty about injected location, targeting accuracy of optrodes, and inability to assess the degree of genetic expression. To alleviate some of these problems, the use of optical windows has permitted clear visualization and targeting of functionally characterized sites, use of fine injection pipettes which do not damage cortical tissue, and repeated optical monitoring of expression. These benefits of optical windows apply for optogenetics studies in both anesthetized and awake animals (Ruiz et al., 2013). Using optical windows, Nassi et al. (2015) have demonstrated that optogenetic stimulation in awake monkey visual cortex can ‘substitute’ for normal visual stimulation in a manner following the divisive normalization model of visual neuronal response and that neuronal response can be parametrically modulated in a controlled fashion. This important advance improves the reliability and accuracy of optogenetics methodology in primates and further secures its role as a focal stimulation modality in non-human primate studies.

#### Functional Tract Tracing with Optogenetic Stimulation

One of the most exciting applications of optogenetics is its use as a behavioral modulation tool and for functional tract tracing. In monkeys, this approach has been used to reveal frontal circuits underlying eye movement behavior. By stimulating ChR2 sites in FEF (the anterior and posterior arcuate sulcus) during performance of target selected eye saccades, Vanduffel and colleagues demonstrated that saccades in trials with stimulation occurred with significantly shorter latency than in those without (Gerits et al., 2012). The shortened saccade latencies suggested that optogenetic stimulation either produced greater neuronal firing rates or recruited a greater number of neurons. The success of this stimulation paradigm was attributed to a few factors: the use of a cell type non-specific CAG promoter, targeted injections in saccade related domains identified with FMRI, and stronger stimulation via the use of dual light guides. Importantly, the assessment of effect was not the induction of eye movement *per se* but rather the facilitation of eye movement latency. Thus, in contrast to early negative results, more finely tuned assessments of behavioral effect can reveal clear, robust effects of optogenetic stimulation. A further and important benefit of this optogenetic approach is its compatibility with the MRI environment. Stimulation of the anterior arcuate in the MR activated loci in V1, V4, MSTd, and MSTv, leading to identification of circuits underlying the speeded eye movement behavior.

At a local scale, optogenetic stimulation has also been used to probe the effect of stimulating single or multiple orientation domains in V1 of the tree shrew (Huang et al., 2014). Rather than use optrodes or fiber optics, this study used a spatial light modulator to produce a pre-specified pattern of blue light to activate specific ChR2 infected orientation columns in the cortex. Surprisingly, they did not find activation of nearby orientation domains of similar selectivity, but rather the effects appeared to fall off with distance from the stimulated domain. This result is not consistent with other results that demonstrate functionally selective effects of optogenetic stimulation via fiber optic delivery in Macaque monkey V1 (Chernov and Roe, 2014b). Stimulation of ocular dominance columns at single sites enhanced (increased reflectance change detected by optical imaging) the stimulated ocular dominance columns (similar to results obtained with INS, **Figures 5D–F**), and stimulation of single orientation domains led to relative enhancement of other orientation domains of similar selectivity. The differences between these studies may relate to intensity or effective depth of stimulation. Further studies are needed to evaluate these effects.

## Conclusion

We have explored three different functional tract tracing methods that can be presented at the columnar scale in a targeted, functionally specific fashion. These methods do not require animal sacrifice and lengthy anatomical reconstruction, and potentially permit the study of connections from a greater number of sites within an individual animal. The similarities and differences between these methods and traditional anatomical tract tracing methods (e.g., in sensitivity to less robust connections) need to be further characterized and understood. While these methods cannot replace the gold standard of anatomical tract tracing, the advantages of *in vivo* functional tract tracing will make studying column-specific brain connections on a large scale more feasible.

The three methods presented each have their strengths and weaknesses. Electrical microstimulation is complicated by current spread but, at least within certain parameters and at a population level, appears to bias the activated circuits in a way that permits visualization of connections via functional imaging methods. Given that there is a wealth of studies demonstrating behavioral modulation using electrical stimulation, the use of electrical stimulation to map connections in the brain provides a direct link to these studies and could be conducted via methods that are already readily accessible. INS stimulation is a new method that is being developed for superficial and deep tissue stimulation. Applied via fiber optics, INS permits focal stimulation. INS does not require the use of viruses, giving it greater potential for human use. However, at this point in time, INS requires the delivery of specific infrared light wavelengths that are not yet commercially available. Optogenetics is a cell specific stimulation method that has revolutionized neuroscience. It requires the introduction of viruses that express light-sensitive rhodopsin molecules. Optogenetics has been enormously useful in studies in mice; its use in monkeys now also looks promising. Given the need for viral transfection, it is less amenable to use in humans.

The promise of conducting *in vivo* functional tract tracing studies calls for new future studies. We propose: (1) targeted, systematic, and large scale evaluation of columnar networks in different functional brain systems in monkeys (e.g., visual system, hand use system, working memory system), (2) cell type specific columnar mapping, (3) direct correlation of behavioral effects and mapped columnar networks, and (4) designing brain-machine interfaces based on targeting of columnar networks.

## Conflict of Interest Statement

The authors declare that the research was conducted in the absence of any commercial or financial relationships that could be construed as a potential conflict of interest.
